# Polygenic Risk Score Associated with Gestational Diabetes Mellitus in an AmericanIndian Population

**DOI:** 10.3390/jpm15090395

**Published:** 2025-08-22

**Authors:** Karrah Peterson, Camille E. Powe, Quan Sun, Crystal Azure, Tia Azure, Hailey Davis, Kennedy Gourneau, Shyanna LaRocque, Craig Poitra, Sabra Poitra, Shayden Standish, Tyler J. Parisien, Kelsey J. Morin, Lyle G. Best

**Affiliations:** 1 Pathology Department, University of North Dakota, Grand Forks, ND 58202, USA; karrah.peterson@und.edu; 2 Natural Sciences, Turtle Mountain Community College, Belcourt, ND 58316, USAtia.azure@tm.edu (T.A.); haileyl.davis1@gmail.com (H.D.); kennedykat87@gmail.com (K.G.); shyanna.larocque@tm.edu (S.L.); craigpoitra@icloud.com (C.P.); sabrarose@outlook.com (S.P.); shaydenbryce@yahoo.com (S.S.); tparisien@tm.edu (T.J.P.); kmorin_2@tm.edu (K.J.M.); 3 Diabetes Unit, Endocrine Division, Massachusetts General Hospital, Boston, MA 02114, USA; camille.powe@mgh.harvard.edu; 4 Broad Institute, Cambridge, MA 02142, USA; 5 Harvard Medical School, Harvard University, Boston, MA 02115, USA; 6 Department of Biostatistics, University of North Carolina, Chapel Hill, NC 27599, USA; quansun@live.unc.edu; 7 Center for Computational and Genomic Medicine, Children’s Hospital of Philadelphia, Philadelphia, PA 19104, USA

**Keywords:** gestational diabetes, genetics, risk score, American Indian

## Abstract

Background/Objectives: Gestational diabetes mellitus (GDM) is a state of hyperglycemia during pregnancy, increasing the risk of birth complications, and subsequent type 2 diabetes mellitus in the mother and offspring. Risk factors such as diet, obesity, and family history have demonstrated strong association with GDM, but no clear pathophysiology has been ascertained. Methods: An analysis was conducted on 38 women with and 296 without GDM, within a case/control study of pre-eclampsia. The genetic variants examined were selected from among a published polygenic risk score of 10 variants (PRS-10). Genetic models were evaluated for each variant by multivariate logistic regression methods adjusted for age, body mass index, and pre-eclampsia. Since the genotypes for three of the PRS-10 were not available, a risk score comprising the total risk alleles among seven of the variants (PRS-7) was evaluated among those with all genotypes available. Results: Multivariate logistic regression showed significant, independent, positive associations between body mass index (BMI) and age. The posited PRS-7 showed a trend (OR 1.56, 95% CI 0.92–2.56, *p* = 0.070), and sensitivity analysis comprising three variants (PRS-3) was significantly associated with GDM (OR 2.43, 95% CI 1.17–5.06, *p* = 0.017). In univariate analysis, rs1421085 was associated with GDM (OR 0.50, 95% CI 0.26–0.95, *p* = 0.034), but not after adjustment for covariates, and paradoxically not for the expected risk allele. None of the other six variants showed an individual association with GDM. The previously published meta-analysis of PRS-10 showed a degree of heterogeneity (*p_Q_* *= 0.03*) among the three cohorts analyzed, suggesting that variant effects may differ according to the genetic background, which points to the importance of examining the generalizability of any posited polygenic risk scores. Conclusions: In conclusion, we provide additional support for and further refine the results of a previously published polygenic risk score for GDM in an ethically unrelated population.

## 1. Introduction

Since 2004, the Genetics and Pre-Eclampsia Study (GPS) of Turtle Mountain Community College has enrolled over 450 pre-eclampsia cases and controls [1,2]. Sufficient genetic and medical record information on associated risk factors, including gestational diabetes mellitus (GDM), was obtained from a subset (N = 334) of participants with identified GDM and a random selection of controls without GDM, to allow for the current analysis.

GDM is a state of hyperglycemia in pregnant women that can be diagnosed as early as 24 weeks of gestation [3]. The consequences of GDM can lead to birth complications such as macrosomia, Cesarean section, increased risk of subsequent type 2 diabetes mellitus (DM-II) in the mother and increased prevalence of DM-II prevalence among offspring [4]. GDM affects approximately 15% of pregnant women [4], but nearby Canadian aboriginal populations have been shown to experience a greater prevalence of GDM than other ethnic groups in the United States [5].

Risk factors such as maternal age, diet, increased body mass index (BMI), and family history have demonstrated strong association with GDM but no clear pathophysiology has been ascertained [6]. Advanced maternal age has been associated with oxidative stress, endothelial dysfunction, and increased inflammation, all of which has been linked to GDM [7]. A low or normal BMI (<30) and nulliparity were “protective factors” against the development of GDM [8,9,10]. Although GDM is a recognized risk factor for pre-eclampsia (PE) [11], whether the reverse is true and whether both are independent of each other is unknown.

Insight into the pathophysiology of GDM has been derived from associations with genetic variants that confer a higher risk of GDM. For example, Powe et al. [12] described a “Pregnancy Cluster 1” (referred to here as “PRS-10”), including the variants listed in Table 1. This PRS-10 was associated with an increased risk of GDM (odds ratio = 1.24, *p*-value = 6.20 × 10^−7^). The present study sought replication, to the extent possible, of this association. Table 1 summarizes PRS-10 variants, their possible mechanistic relationship with GDM and the subset analyzed in the current report.

## 2. Materials and Methods

### 2.1. Ethics Approval and Participants

Written informed consent was obtained from all participants permitting the analysis of potential genetic and other PE risk factors, including GDM. Three independent Institutional Review Boards (IRBs) authorized the GPS study, including the Great Plains Indian Health Service (02-R-11AA, 4/22/2005), the Tribal Nations Research Group (protocol #39, 2/18/2010), and the University of North Dakota (IRB-200207-001, 8/13/07). All participants qualify for Indian Health Service benefits which require the demonstration of American Indian inheritance, except for uncommon instances where only the father of the child is of American Indian heritage. Approval was also obtained from the participants’ Tribal governments, who generally disapprove of genetic comparisons between Tribes and other populations.

The above-referenced GPS dataset and samples were accessed to conduct the present analysis. The analysis cohort of each variant aimed to include all participants with the identified GDM and a convenience sample of randomly selected controls with available samples to genotype, or pre-existing genotypes (from a previous microarray). Genotyping assignments to student staff resulted in differing numbers of cases and controls for each of the variants. No formal power analysis was conducted, but obtaining somewhat over 100 genotypes for each variant was considered sufficient to begin analysis.

In the prior GPS analyses, investigating genetic associations with pre-eclampsia, gestational diabetes mellitus (GDM), and pre-existing diabetes were included as covariates, though the primary focus was on PE. Data on these conditions were abstracted from medical records or birth certificates by one of the authors or a supervised laboratory assistant. GDM was defined as the presence of a clinical diagnosis of “gestational diabetes” or “glucose intolerance” during pregnancy, without prior history of diabetes. Some cases of GDM may have been missed due to incomplete records, as the GPS did not specifically focus on diabetes. Participants with a history of diabetes prior to pregnancy were excluded.

Information on important co-variants, such as age at delivery, nulliparity, BMI (calculated from weight at first prenatal visit), and documented PE during the pregnancy were available from the GPS dataset. The diagnosis of PE was consistent with previously published GPS methods [1] and required at least 2 of 3 criteria reflecting hypertension (three blood pressures over 140 systolic or 90 diastolic), proteinuria (2 or more urinalyses with 1+ protein or greater) and a clinical diagnosis of PE. Covariate information was complete for all 334 participants.

### 2.2. Genotyping

Salivary samples were collected and processed according to manufacturer’s protocol (DNA Genotek Inc., Stittsville, ON, Canada, OGR-500). Quality assessment utilized a 260/280 nm ratio in the range of 1.60–1.90, provided by a NanoDrop (ThermoFisher Scientific Inc., Waltham, MA, USA) instrument.

Genotypic data from an Illumina Infinium microarray (ITMAT-Broad-CARe, IBC, version 1) [20] was available for 2 variants, rs1421085 and rs10830963, (please see the reference for procedural details). Samples for microarray genotyping were standardized to 5 ng/uL and QC standards at The Children’s Hospital of Philadelphia were monitored with a mean call rate above 98% for all variants and less than 4% of all samples with a call rate below 95%. The imputation of missing microarray genotypic data was not utilized, primarily due to uncertainty as to whether standard population datasets would provide accurate results.

Four variants were genotyped by a TaqMan assay (ThermoFisher Scientific Inc., Waltham, USA, Catalog#4351379, rs523288:C____797207_10, rs917195:C___7597950_10, rs4976033 C__27946268_10, rs13085136:C____266223_10). Salivary DNA was extracted as noted, and approximately 350 ng of template used in a 20 uL reaction volume of diluted TaqMan Genotyping Master Mix, Applied Biosystems, Waltham, MA, USA, Catalog #4371355). Reactions were carried out on a BioRad MiniOpticon (BioRad Laboratories Inc., Hercules, CA, USA) real-time PCR platform, using the standard thermocycling program. Genotypes were assessed by visual inspection using Bio-Rad CFX Manager 3.1 software. All TaqMan assay runs were conducted with a blank and known samples representing the 3 genotypes. A TaqMan assay was also used to replicate the microarray genotyping results and allele designation for rs1421085 with confirmation on 43 of 44 samples.

One variant, rs10954772, was assessed by targeted Sanger sequencing after a custom TaqMan assay (ThermoFisher Scientific Inc., Waltham, USA, Catalog # 4351379) failed. In this instance, the ThermoFisher custom TaqMan assay did not provide any discernable genotypic discrimination on approximately 30 samples and the company was unable to provide any alternative reagents or advice to resolve the problem. Thus, a 612 bp amplicon was generated using LongAmp *Taq* DNA Polymerase (New England Biolabs, Ipswich, MA, USA, Catalog #MO323S) using forward (TTGAATGCCAGTGTCTGGGAG) and reverse (ATAGGTGGATTTGGGACAGCA) primers and a thermocycler protocol consisting of 94 C (20 s), 62 C (40 s), and 65 C (50 s) over 30 cycles, with a final 10 min at 65 C. The amplicons were purified with QIAquick PCR Purification Kit (Qiagen, Hilden, Germany, Catalog #28104). Big Dye Terminator 3.1 (ThermoFisher Scientific Inc., Waltham, USA, Catalog # 4337455) reactions were run and products cleaned using BigDyeXTerminator (ThermoFisher Scientific Inc., Waltham, USA, Catalog #4376486), according to the manufacturer’s recommendations. Capillary electrophoresis was conducted on a SeqStudio instrument (ThermoFisher Scientific Inc., Waltham, USA, Catalog # A35644). The SeqStudio base called output (Sequencing Analysis Software, ThermoFisher Scientific Inc., Waltham, USA, Catalog #38938, V7.x) was opened in open-source Unipro UGENE (v33.0) and genotype interpreted directly from the electropherogram after searching for the unique 7 bp sequence just prior to the variant. “Functionally tested” ThermoFisher TaqMan assays were unavailable for the remaining SNPs shown in Table 1.

### 2.3. Statistical Analysis

A pre-study power analysis was not conducted and not required for IRB approval, since this was a secondary analysis of specimens collected for the study of pre-eclampsia. SPSS v13.0 was utilized to run all statistical analyses. Descriptive statistics show means (SD) for quantitative traits and N (%) for discrete variables. Tests of statistical significance utilized Chi-square and the T-test of independent means for discrete and continuous variables, respectively. Multivariate logistic regression models included age at delivery, BMI, nulliparity, and PE. Since nulliparity was not significant in univariate analysis, it was not included in the multivariate model.

To avoid confounding from potential population stratification, a principal components analysis (PCA) of the microarray genotypes was conducted [21]. The 45,554 IBC SNPs with rsID designation were filtered to exclude the 7 variants included in the risk score, any failing to genotype in any sample, those with a minor allele frequency less than 0.01, and those exhibiting linkage disequilibrium of r^2^ > 0.10. There were 8655 SNPs remaining in the PCA analysis and the top 10 principal components (PCs) were entered into the multivariate model. The odds ratio and 95% confidence intervals are reported, and statistical significance was evaluated at the *p* = 0.05 level. While recruitment occurred at different times among 3 different Tribes, only one (representing 96% of the total) had genotypic microarray data, and thus contributed to the PCA analysis.

In the partial replication of Powe et al. [12], the 7 available genotypes were used to create the present PRS-7. The choice of these genotypes was random, in so far as they were available as standard, ThermoFisher TaqMan assays or happened to be included in a previous microarray analysis of PE for the GPS. The only non-random choice was that of rs10954772 for sequencing, which was selected from the unavailable genotypes because it had the greatest weight among the variants in this cluster. The sequencing of the other 3 genotypes without available data would be very time-consuming and thus analysis of the available 7 genotypes was undertaken. This score (PRS-7) was a summation of the risk alleles available (Table 1) for each participant, limited to those with genotypic data for all 7 variants. Unfortunately, it was necessary to limit the analysis to the 99 samples with complete genotypic data, since there was a significant excess of missing genotypes among controls compared to cases. The distribution of PRS-7 was from 0 to 10 from a possible total of 14. A sensitivity analysis was carried out, selecting 3 (PRS-3) of the PRS-7 genotypes that showed a single variant, adjusted odds ratios > 1 (including rs523288, rs10830963, and rs4976033).

## 3. Results

### 3.1. Characteristics of the Cohort

The case, control, and covariate distributions shown in Table 2 reveal expected differences between those with GDM and those without, such as the mean age (28 vs. 24 years), BMI (35 vs. 29), prevalence of nulliparity (42% vs. 51%), and PE (58% vs. 40%), respectively. Although different for each variant, the prevalence of GDM in the overall analysis cohort is 11.4% and approximates typically reported values [4]. Risk allele frequencies in the complete cohort are found in Table 3 and generally comparable to the reported TOPMED frequencies [22].

### 3.2. Univariate Associations

The univariate logistic regression results of both the covariates and the genetic variants are shown in Table 4. Only the results of those genetic models (e.g., additive or dominant) with the smallest *p*-values were displayed, and that model continued to be used in the subsequent analyses. As might be expected, maternal age, BMI, and PE showed an association with GDM, whereas the only genetic variant, rs1421085, in a C-additive model, was nominally associated. None of the principal components showed a significant association with GDM, although PC 7, 8, and 10 are likely unreliable due to the low frequency of component SNPs. Figure S1 in the Supplementary Materials shows the plots of PC1/2 and PC2/3.

### 3.3. Multivariate Associations

The results of a fully adjusted model of the covariates are found in Table 5. We found that this multivariate adjustment attenuates the association of PE, but demonstrates the independent contribution of both BMI and maternal age. The principal components are not significantly linked to GDM and in a number of cases (e.g., PC-7 and PC-10) are not credible, as noted above.

When genetic variants were individually added to the above fully adjusted model, none reached the level of significance. The univariate, nominal association of rs1421085, C-ADD with GDM lost significance in the fully adjusted model (*p* = 0.162).

### 3.4. Polygenic Risk Scores

Limited to the subset with the full complement of genotypes, the PRS-7 score is a cumulative total of an individual’s available risk alleles and shows a trend toward association with GDM, after multivariate adjustment (*p* = 0.070). The distribution of PRS-7 was from 0 to 10 from a possible total of 14, as seen in Table 6.

A sensitivity analysis reducing the components to the three genetic variants with positive odds ratios (PRS-3, including rs523288, rs10830963, and rs4976033) demonstrated a significant association in both adjusted and unadjusted analyses. An alternative PRS-7 utilizing the weighting scores of Powe et al. [12] was also tested, but did not yield significant results.

**Figure 1:**
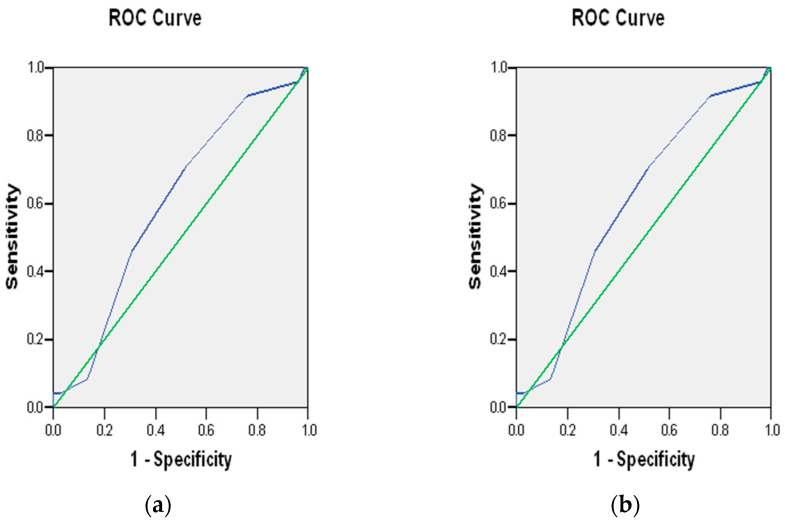
Receiver operating characteristic plot of (**a**) PRS-7 and (**b**) PRS-3. The green line indicates the baseline, “null” plot, the blue line shows the experimental results.

**Figure 2:**
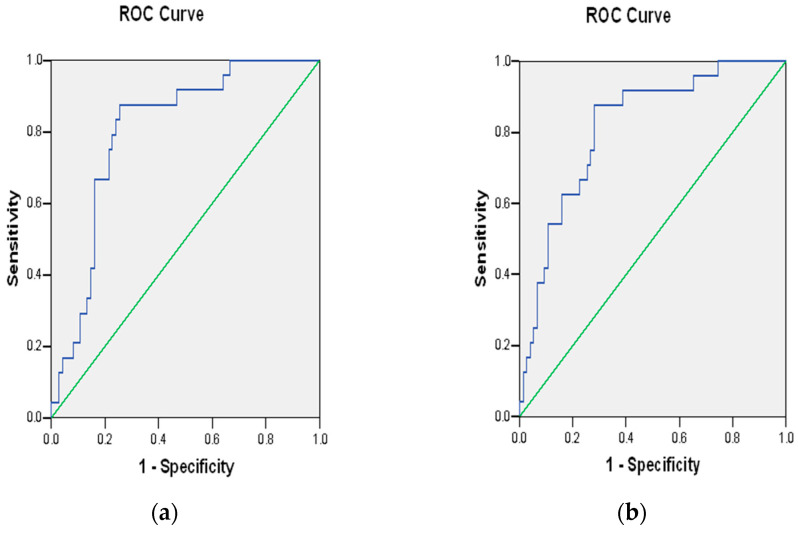
Receiver operating characteristic plot of (**a**) age x BMI and (**b**) age x BMI x PRS-3. The green line indicates the baseline, “null” plot, the blue line shows the experimental results.

The receiver operating characteristic (ROC) plots of PRS-7 and PRS-3 are shown in Figure 1. There appears to be enhanced performance for PRS-3, but both have areas under the curve (AUC) of 0.601 and 0.644, respectively, and values in this range are generally not clinically useful. Comparing an age*BMI interaction term with and without PRS-3 is shown in Figure 2, and the addition of PRS-3 appears to increase the AUC slightly, from 0.804 to 0.814. Considering the potential to “rule out” GDM, or at least reduce the need for prenatal glucose tolerance testing, we modeled the lack of even a single PRS-3-risk allele (N = 21) to predict the absence of GDM. This showed a poorly delimited odds ratio estimate of 6.33 (95% CI 0.55–72.45, *p* = 0.138) and an ROC AUC of 0.613.

## 4. Discussion

Among a subset with complete genotypic data, a multivariate analysis adjusted for age and BMI, utilizing a subset (PRS-3) of a previously reported polygenic risk score found a significant association with GDM (OR 2.43, 95% CI 1.17–5.06, *p* = 0.017). Age at delivery and BMI were also independently linked to an increased risk of GDM. Despite the association of the *FTO*, rs1421085 C allele with an increased BMI and GDM risk in the literature, in the present analysis, the C allele was found to confer a lower univariate risk (but not after adjustment in the multivariate model).

### 4.1. Rationale for Variants Included in the Analysis

We examined a selection of 7 SNPs among the 10 genes associated with GDM in the literature [12]. These SNPs were variants of *MC4R*, *PURG*, *CRHR2*, *FTO*, *MTNR1B*, *PIK3R1*, and *SHQ1*, and were chosen from Powe et al.’s “Pregnancy Cluster 1” [12], after consideration of the TaqMan assay and previous microarray genotype availability. The essentially random choice of these 7 variants from the original 10 would seem to preclude any significant bias in comparing the earlier report and these results. The above-referenced study [12] used a sophisticated statistical approach to cluster groups of putative genetic variants according to probable physiologic function, which were then assessed for association to GDM. These SNPs were related to the risk of GDM, type 2 diabetes mellitus, and/or reduced insulin sensitivity in other studies as well [18,23,24].

### 4.2. Functionality of the Variants Included

The *MTNR1B* gene SNP has been among the most intensely studied genes related to glucose homeostasis in pregnancy [16]. *MTNR1B* encodes for melatonin receptor 1B binding melatonin, which reduces insulin secretion from pancreatic beta cells. The presence of the G allele of this variant increases the expression of melatonin receptor 1B and increased melatonin binding, resulting in low insulin secretion [25]. A meta-analysis of 8 cohorts (3296 cases and 3709 controls) found an odds ratio of 2.228 (95% CI 1.224–4.055, *p* = 0.009) modeling an rs10830963 G recessive genotype on GDM risk [23]. The present analysis showed the same direction of effect but was not statistically significant, perhaps due to a lack of power, although this variant is favorably powered in comparison to others in this study.

The *PIK3R1* gene plays a crucial role in regulating insulin signaling pathways [26] by encoding a key regulatory subunit interacting with insulin receptor substrates (IRS1/2). The binding of p85alpha (produced by *PIK3R1*) to IRS1/2 triggers downstream effects, including increasing GLUT4 at the cell membrane, stimulating glycogen synthesis, and suppressing gluconeogenesis [17]. A study examining insulin sensitivity indices and gene variants affecting these indices found that *PIK3R1* gene rs4976033 variant was associated with changes in glucose levels during an oral glucose tolerance test (OGTT) at 0, 30, and 120 min suggesting a potential role in reducing insulin sensitivity [24].

*MC4R* encodes the melanocortin 4 receptor, helping regulate satiety and hunger either by its gain or loss of function [27]. The gain of function increases satiety while loss leads to overeating and eventually obesity. Although the direction of effect noted in the current study is consistent with the literature, there is probably insufficient power to demonstrate an association. We were also unable to detect a significant linear regression association between this risk allele and BMI, although the direction of effect was as expected (beta 1.48, SE 1.49, *p* = 0.325).

The FTO gene SNP rs1421085 may indirectly influence GDM development by increased maternal adiposity. The CRISPR–Cas9 editing of the T-to-C allele of this variant in adipocytes causes increased expression of IRX3 and IRX5 genes and shifts the function of the cell toward that of fat storage and reduced mitochondrial thermogenesis [28]. Saucedo et al. also found the risk (C allele) is associated with increased weight gain in pregnancy, as well as increased adiponectin and TNF-alpha levels [29].

### 4.3. Implications of Individual Variant Results

Among the seven SNPs reported here, only rs1421085 was individually associated with GDM in univariate analysis (Table 4). The finding that the C allele associated with reduced risk in this cohort, in contrast to the literature, is difficult to explain. We checked the direction of effect repeatedly and conducted replicate genotyping of multiple samples with a TaqMan assay to confirm the microarray designation of alleles. We were unable to postulate any environmental influences in this population that could explain this, and feel we adequately accounted for potential population stratification. Although highly speculative, given the previously noted secondary effects of this allele on the expression of IRX3 and IRX5, it is possible that there unrecognized variants of these latter genes in the current population that modify the effects of rs1421085.

Further analysis of our cohort failed to provide evidence for any association between the other six SNPs evaluated, either through univariate or multivariate logistic regression models adjusting maternal age at delivery and BMI.

### 4.4. Implications of Polygenic Risk Score Results

The imbalance of missing genotypes between cases and controls, unfortunately, limited our sample size further to N = 99 (24 cases and 75 controls) when we were required to limit analysis to those with all seven genotypes available. In spite of this, PRS-7 showed a trend toward significance and the PRS-3 sensitivity analysis was significant in both univariate and multivariate analyses.

The fact that PRS-7, comprising the total number of alleles reportedly contributing to risk from these, showed even a marginal association with GDM was somewhat unexpected, especially since four of this group exhibited trends in conflict with the anticipated risk allele (albeit only rs1421085 was statistically significant). While our sample size is certainly small, it is worth noting that one component of a meta-analysis (TGDM-NDM) with only 86 cases and 180 controls, and a four-variant PRS, showed an odds ratio of 1.68, 95% CI 1.23–2.29, *p* = 0.001 [30].

Since only three of the SNPs in PRS-7 showed association with GMD in the expected direction of effect, we conducted a sensitivity analysis of this group of variants alone (PRS-3), demonstrating a significant adjusted odds ratio (OR 2.43, 1.17–5.06, *p* = 0.017). The results of those variants with unexpected direction of effect may be explained by the relatively low power of a number of these analyses; however, the results of an early PRS report are also instructive [31]. This study was of similar design, but with a much larger cohort (N = 1996); among 34 variants comprising the PRS, the odds ratio point estimate was positive in only 24, and only 8 showed a nominally significant association with GDM. Nonetheless, the PRS indicated a highly significant association with GDM (odds ratio 1.10, 95% CI 1.07–1.13, *p* = 6 × 10^−11^). We also considered a modified PRS-7 using the weighting scores derived by Powe et al. [12] and found no improvement.

We identified nine studies reporting the association of various PRS with GDM, with four populations in China [30,32,33,34], three of primarily European heritage [31,35,36], one South Asian [37], and one international collaboration [12]. Of these, seven incorporated no more than two variants from PRS-7 and the remainder had no variants in common, thus making direct comparison of results difficult. Generally speaking, the added contribution of the PRS to models, including maternal age and BMI, provided very modest improvement, with the greatest showing an AUC increase of 0.675 to 0.753 [30], and the lowest from 0.67 to 0.70 [31].

### 4.5. Limitations

Although the genetic homogeneity of the current study population is likely greater than most published analyses, and adjustment for principal components was re-assuring, there always remains a possibility that residual population stratification may exist. The sample size was small, but there was a nominally significant association. While our results for PRS-3 are statistically significant, in ROC analysis, the increased AUC derived from its addition to the main predictors of maternal age and BMI is small and not statistically significant. Thus, the clinical utility of PRS-3 is unlikely.

## 5. Conclusions

The current study aimed to analyze the gene variants associated with the development of GDM, and applied it to our smaller Native American cohort. With a total of 334 participants, our cohort provided a limited but reasonable dataset for statistical analysis in this community. We were able to demonstrate results consistent with that of different populations [12,31,33,34,35,36,37]. The strength of association between GDM and a more limited PRS-3 was unanticipated, especially given the lack of significant results when evaluating each SNP individually. Unfortunately, although statistically significant, the effect size of PRS-3 is limited and there appears to be little clinical utility. The confirmation of the relationship of BMI and age at delivery in this population was reassuring. Interestingly, the *FTO* risk allele analyzed was protective against GDM in the present, univariate analysis.

GDM plays a significant role in maternal and neonatal health outcomes, thus warranting continued research for improved PRS. The ability to better detect the propensity for developing GDM is a useful diagnostic tool that could potentially reduce prenatal testing burden, and enhance management or aid in the prevention of GDM in the future.

## Figures and Tables

**Table 1:** PRS-10 genetic variants and those in the current analysis.

Gene	SNP *	Risk/Alternate Allele	Included in Current Analysis	Theorized Mechanism
*MC4R*	rs523288	T/A	+	Obesity [12,13,14]
*PURG*	rs10954772	T/C	+	Adiposity [12,15]
*CRHR2*	rs917195	C/T	+	Pancreatic beta-cell dysfunction [12]
*FTO*	rs1421085	C/T	+	Obesity [12,13]
*MTNR1B*	rs10830963	G/C	+	Insulin resistance Pancreatic beta-cell dysfunction [12,16]
*PIK3R1*	rs4976033	G/A	+	Insulin resistance [17]
*SHQ1*	rs13085136	C/T	+	Adiposity [12,18]
*MRPS30*	rs6884702	G/A		Unknown [12]
*GLP2R*	rs7222481	C/G		Pancreatic beta-cell dysfunction [12,19]
*SLC2A2*	rs9873618	G/A		Hepatic glucose uptake [12]

* Single-nucleotide polymorphism.

**Table 2:** Case–control characteristics.

	GDM	Control	*p* Value
Number (N)	38	296	
Age at delivery mean (SD)	28.0 (6.48)	23.8 (5.73)	3 × 10^−5^
Parity, N (% nulliparous)	16 (42.1%)	151 (51.0%)	0.301
Body mass index (SD)	34.8 (8.10)	28.7 (7.15)	1.4 × 10^−6^
Pre-eclampsia, N (% yes)	22 (57.9%)	117 (39.5%)	0.031

**Table 3:** Frequency of the risk alleles and assessment of the Hardy–Weinberg equilibrium.

	Risk Allele *	Case Risk Allele Frequency (%)	Control Risk Allele Frequency (%)	Case vs. Control Risk Allele Frequency ** p Value	Hardy-Weinberg *p* Value ***
rs523288	T	11/66 (16.7)	29/230 (12.6)	0.518	0.621
rs10954772	T	19/70 (27.1)	96/312 (30.8)	0.650	0.812
rs917195	C	34/50 (68.0)	170/236 (72.0)	0.689	0.920
rs1421085	C	13/76 (17.1)	158/554 (28.5)	0.050	0.361
rs10830963	G	22/72 (30.6)	151/538 (28.1)	0.764	0.679
rs4976033	G	30/50 (60.0)	134/216 (62.0)	0.916	0.871
rs13085136	C	42/48 (87.5)	240/272 (88.2)	0.923	0.091

* as per Powe et al. [12], ** Pearson Chi-square with continuity correction, *** including both the cases and controls.

**Table 4:** Univariate logistic regression results (additive, and both dominant genetic models were tested for each variant, and the result with the lowest *p*-value is shown for brevity).

	Risk/Alt Allele *	Odds Ratio	95% Confidence Interval	*p*-Value
Age at delivery		1.114	1.06–1.17	<0.001
Nulliparity		0.698	0.35–1.38	0.303
Body mass index		1.093	1.05–1.14	<0.001
Pre-eclampsia		2.104	1.06–4.18	0.033
rs523288, T-ADD	T/A	1.408	0.65–3.06	0.388
rs10954772, T-Rec	T/C	0.240	0.03–1.87	0.173
rs917195, C-Dom	C/T	0.606	0.15–2.42	0.478
rs1421085, C-ADD	C/T	0.499	0.26–0.95	0.034
rs10830963, G-Rec	G/C	1.403	0.45–4.33	0.556
rs4976033, G-Dom	G/A	1.131	0.46–2.79	0.789
rs13085136, C-ADD	C/T	0.923	0.34–2.52	0.876
PRS-7 **		1.266	0.92–1.74	0.144
PRS-7, weighted *		1.201	0.89–1.62	0.232
PRS-3 ***		1.673	1.08–2.59	0.021

* as per Powe et al. [12], ** both PRS-7 and PRS-3 results derived from 24 cases and 75 controls with complete genotypic data, *** a subset of PRS-7 comprised of the three genotypes with odds ratios > 1 in univariate analysis.

**Table 5:** Multivariate logistic regression results (additive, and both dominant genetic models were tested for each variant, and the result with the lowest *p*-value is shown for brevity).

	Risk/Alt Allele *	Odds Ratio	95% Confidence Interval	*p*-Value
Age at delivery		1.099	1.03–1.18	0.006
Body mass index		1.079	1.02–1.14	0.005
Pre-eclampsia		2.011	0.81–5.01	0.134
PC-1 **		0.032	0.00–146.8.8	0.423
PC-2		0.027	0.00–3432	0.548
PC-3		0.018	1.03–128.9	0.375
PC-4		0.152	0.00–2597	0.704
PC-5		0.299	0.00–3853	0.802
PC-6		0.001	0.00–115.7	0.230
PC-7		0.598	0.00–52,172	0.929
PC-8		0.276	0.00–10,894	0.812
PC-9		1.378	0.00–1310	0.927
PC-10 ***		13,152	0.96–179,234,369	0.051
The above covariates included in analysis with each of the following independently				
rs523288, T-ADD	T/A	1.523	0.51–4.55	0.451
rs10954772, T-Rec	T/C	0.970	0.84–11.15	0.981
rs917195, C-Dom	C/T	0.375	0.06–2.46	0.306
rs1421085, C-ADD	C/T	0.613	0.28–1.35	0.225
rs10830963, G-Rec	G/C	1.822	0.45–7.40	0.402
rs4976033, G-Dom	G/A	1.330	0.36–4.85	0.666
rs13085136, C-ADD	C/T	0.743	0.22–2.46	0.627
PRS-7 ****		1.569	0.96–2.56	0.070
PRS-7, weighted *		1.437	0.92–2.26	0.114
PRS-3 *****		2.436	1.17–5.06	0.017

* as per Powe et al. [12]. ** principal component 1, etc. *** results considered unreliable, likely due to the low frequency of component SNPs. **** all PRS results derived from 24 cases and 75 controls with complete genotypic data. ***** a subset of PRS-7 comprised the three genotypes with odds ratios > 1 in univariate analysis.

**Table 6:** Distribution of PRS-7 scores among those with complete data (N = 99).

PRS-7		
Score	Number with Score	%
0	1	1.0
1	3	3.0
2	16	16.2
3	23	23.2
4	22	22.2
5	22	22.2
6	9	9.1
7	2	2.0
8	1	1.0
9	1	1.0
10	3	3.0
PRS-3		
0	21	21.2
1	29	29.3
2	33	33.3
3	11	11.1
4	5	5.1

## Data Availability

The data underlying the results of this study are owned and controlled by the Tribes that approved its collection. This fact is clearly stated in the Tribal resolutions authorizing the research; and it must be recognized that these Tribal communities are independent, sovereign governments, in control over research activities within their borders. Access to data and materials is accomplished by application to Ms Anita Frederick, President of Tribal Nations Research Group, 717 Chief Little Shell St, Belcourt, ND 58316, 701-477-5526 or tribalnationstnrg@gmail.com, who will arrange for further consultation with the appropriate Tribal officials. Approximately 2-to-3 months may be required. The authors received special access privileges to the data due to their relationship with the Tribal Government; however, interested researchers who apply for data access will be able to access the same data as the authors.

## Supplementary Materials

The following supporting information can be downloaded at: https://www.mdpi.com/article/10.3390/jpm15090395/s1, Figure S1: Principal Component plots of (a) PC1/PC2 and (b) PC2/PC3.
