# Supplementary figures and images for: Polygenic Risk Score Associated with Gestational Diabetes Mellitus in an AmericanIndian Population
Source: J Pers Med. 2025 Aug 22;15(9):395. doi: 10.3390/jpm15090395 (PMC12470582; doi:10.3390/jpm15090395)

Figure S1: Principal Component plots of (a) PC1/PC2 and (b) PC2/PC3.

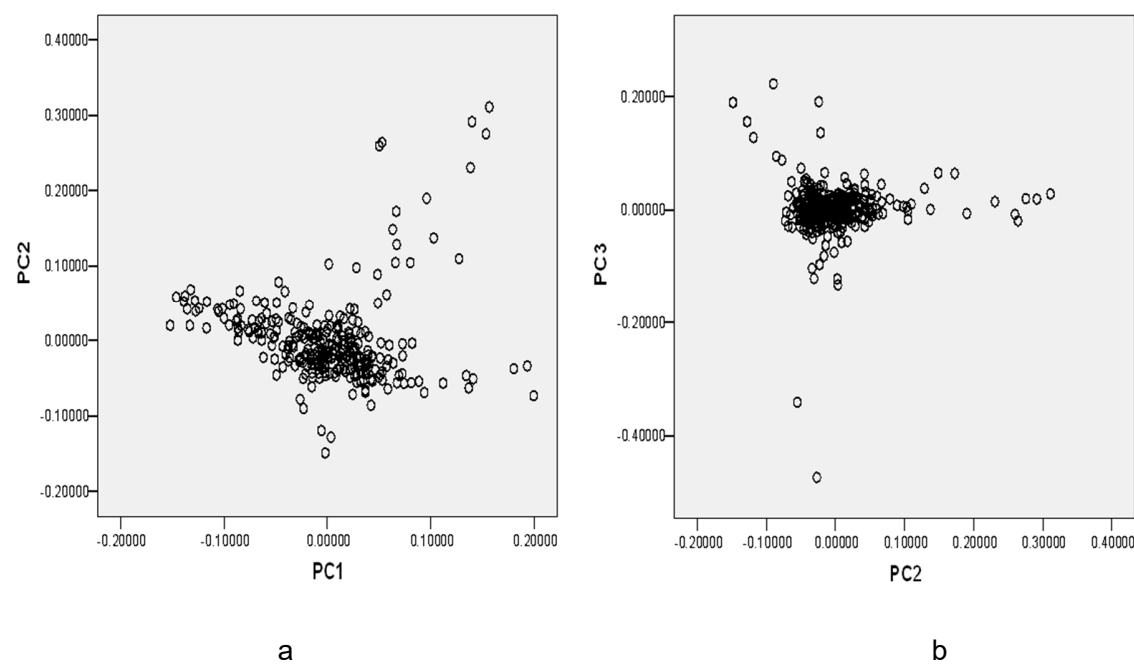

Supplement: Supplementary file 1 [file jpm-15-00395-s001.zip › jpm-3740275-supplementary.pdf]
